# Analysis of the Indian Government’s position on the use of asbestos and its health effects

**DOI:** 10.5588/pha.23.0013

**Published:** 2023-06-21

**Authors:** R. Singh, A. L. Frank

**Affiliations:** 1 Department of Architecture, School of Planning and Architecture, New Delhi; 2 Tathatara Foundation, Bobbili, India; 3 ISAC Centre for Built Environment Policy, Delhi National Capital Region, India; 4 Public Health, Medicine, (Environmental & Occupational Health), and Civil, Architectural & Environmental Engineering, Drexel University, Philadelphia, PA, USA

**Keywords:** chrysotile, mesothelioma, India, executive stand, cancer

## Abstract

Based on WHO guidance, all forms of asbestos are a health risk. In India, the mining of asbestos has been stopped, but chrysotile (a type of asbestos) is still imported and processed in large quantities. Chrysotile is mainly used for asbestos-cement roofing, and the manufacturers claim its use to be safe. We sought to understand the Indian Government’s position on the use of asbestos. To do so, we have analysed the replies of the executive wing of the Indian Government to questions on asbestos in the Indian Parliament. This revealed that, despite a mining ban, the government has defended the import, processing and continued use of asbestos.

## Abstract (fr)

Selon les directives de l’OMS, toutes les formes d’amiante présentent un risque pour la santé. En Inde, l’extraction de l’amiante a été arrêtée, mais le chrysotile (un type d’amiante) est encore importé et transformé en grandes quantités. Le chrysotile est principalement utilisé pour les toitures en ciment-amiante, et les fabricants prétendent que son utilisation est sûre. Nous avons cherché à comprendre la position du gouvernement indien sur l’utilisation de l’amiante. Pour ce faire, nous avons analysé les réponses de l’aile exécutive du gouvernement indien aux questions sur l’amiante posées au Parlement indien. Cela a révélé que, malgré l’interdiction de l’extraction minière, le gouvernement a défendu l’importation, la transformation et l’utilisation continue de l’amiante.

According to the WHO, all forms of asbestos are a health risk.^1^ Despite a ban imposed on the mining of asbestos in India, a type of asbestos called chrysotile is imported, processed and used, most often by the construction industry for asbestos-cement roofing. We therefore sought to understand the position of the Indian Government on the use and health effects of asbestos. To do so, we analysed the replies of the executive wing of the Indian Government (comprising the Ministries and the Cabinet) given in the Indian Parliament.

We present the views of the Indian Government with respect to its stance on asbestos, its use and its health effects. This analysis is based on quoted replies in the public domain and may be used as the basis for any policy study on the use of asbestos in India. To ensure neutrality and lack of bias, the study was based on the list of questions in Parliament taken from the website of the Association of Asbestos Cement Products (New Delhi, India), which advocates for the continued use of asbestos.^2^ The answers were compiled verbatim and are presented along with the questions asked. When applicable, the most recent position of the government has been reported in chronological order, or it has been reported with the year of the reply.

In response to a recent question on the position of the government on types of asbestos being used, procured or banned, the Ministry of Chemicals and Fertilisers of the Indian Government stated that it recognised six varieties of asbestos: crocidolite, actinolite, anthophylite, amostile, tremolite and chrysotile.^3^ It also recognises the Rotterdam Convention, which lists crocidolite, actinolite, anthophyllite, amosite and tremolite in Annexure III (the list of hazardous chemicals and pesticides). This makes the prior consent procedure mandatory by any exporting country when supplying any material listed to India. Annexure III excludes chrysotile,^3^ which is not included because the Indian Government states that ‘most of the asbestos industry in India follows the wet process, which minimises the dispersion of fibres in the air’.^3^ The WHO has stated that all forms of asbestos cause lung cancer, mesothelioma, cancer of the larynx and ovary and asbestosis (fibrosis of the lungs).^1^ This exposure to fibres includes the working environment in the vicinity of the sources (such as factories and mines) and also within buildings containing crumbling asbestos materials.^1^ To date, chrysotile has not been banned in India, and nor has its import been restricted. This was the Indian Government’s response in 2013 when asked whether the Indian asbestos market had grown.^4^ According to the Minister of Mines in 2017, the Minister of Environment in 2016 and the Minister of Chemicals in 2011, the government stated that India imported large quantities of asbestos for asbestos-cement pipes, roofing for households, brake linings, etc., from Russia, Kazakhstan, Brazil, the People’s Republic of China and Canada. The government was questioned about the import of white asbestos and asked whether it proposes to curb the import of the ‘deadly material of asbestos’, whether white asbestos was still being used in India, whether the Union Government was giving due importance to the serious health hazards in the production of asbestos and whether it is still widely used as a material for roofing, flat sheets, cement pipes, etc.^5–7^

Responding to the most recent question posed in 2022, the Ministry of Environment, Forests and Climate Change reiterated the Government’s position that there was no proposal to prohibit the use of asbestos in the country.^8^ This is despite the Indian Government’s own notification under Section 25(1) of the Mines Act, 1952, which recognises the diseases that can be caused by asbestos. These include asbestosis and cancer of the lung or the peritoneum or pleura (i.e., mesothelioma). This information was provided by the Minister of Mines in response to the question on the number of mines and details of health risks associated with asbestos mining.^9^ Despite the WHO warning about the dangers of chrysotile, some manufacturers continue to claim that it is a safe product in their annual business compliance reports.^10^ However, studies in other countries have revealed that even with strict control, asbestos cannot be handled in a safe manner.^11^ In 2011, when asked whether ‘asbestos despite being hazardous to patients and the environment is being used by industries in the country’, the Ministry of Health stated (as per the Parliamentary records) that the health risks of the use of chrysotile asbestos were yet to be proven.^12^ In 2005, a legislator voluntarily raised awareness about the health risks associated with asbestos by citing a study published by an Indian physician in Parliament.^13^ The study concluded that asbestos, in any form and under any circumstances, is carcinogenic and that there are no safe levels of exposure, a position supported by numerous international agencies. In response, the government issued a rebuttal stating that its agency, the Indian Council of Medical Research (New Delhi, India), had found that there was no convincing evidence that consumers of asbestos products faced any significant risk of cancer.^13^ In many countries, there is documented evidence to suggest that the consumer use of asbestos products is linked to disease.^14^ In 2009, the Human Rights Commission of Kerala, India, banned the use of asbestos roofing for schools,^15^ and this was followed by an injunction by the Calcutta High Court on its use in the construction of the court complex.^16^ To further study the environmental and health effects of chrysotile in India, the Ministry of Chemicals and Fertilisers commissioned a study entitled “Study of health hazards/environmental hazards resulting from use of chrysotile variety of asbestos in the country”,^17^ carried out by the National Institute of Occupational Health of Indian Council of Medical Research, Ahmedabad. An inter-ministerial study of the various ministries in India evaluated the report and concluded that there was no indication of any significant health/environmental hazard resulting from the use of chrysotile asbestos under normal conditions. In 2017, the environment minister was questioned about the Indian Government’s plans to ban the use of asbestos-based products due to estimates linking asbestos to millions of occupational deaths worldwide. In response, he cited the wet process being used by asbestos product manufacturing units in the country, and the cost-effectiveness of the material, as reasons for its continued use. He also emphasised that asbestos was widely used by the masses.^18^ However, in 2014, the Indian Government relied on a study by the same institute that clearly stated that all types of asbestos were responsible for human mortality and morbidity, and that workers exposed to higher workplace concentrations of asbestos fibres had a higher incidence of interstitial lung disease and pulmonary function impairment. This was in response to a question as to whether asbestos can lead to various life-threatening diseases, including lung cancer, etc.^19^ Despite a 2008 study conducted by the Central Pollution Control Board titled “Human Health Risk Assessment Studies in Asbestos-Based Industries in India,” which confirmed that all types of asbestos cause human mortality and morbidity, there are concerns about the scientific integrity and independence of other studies. There may also be instances of incomplete recording of mesothelioma cases due to the disease’s high latency period.^20^ This study indicated that a population exposed to asbestos fibres showed a marked increase in the deterioration of lung function compared to a control population not exposed to asbestos fibres. In 2013, the Ministry of Mines was also asked in Parliament whether the study examined the effect of asbestos mining on labour health.^21^ The Indian Government faced civil society pressure and public interest litigation on the matter, prompting the Supreme Court of India to hear two cases, one in 1995 and the other in 2011.^22,23^ The court’s orders in both cases are relevant to this issue. These, and other actions by civil society, led the Government to take the following actions:

1. The Factories Act of 1948 recognised the hazards of manufacturing, handling and processing asbestos, and specific model rules were developed for asbestos.
2. In 2017, in response to a question regarding white asbestos and its increasing import to India, regulations were put in place to limit airborne asbestos levels to the permissible limit.
3. The mining of asbestos was banned by not issuing new licenses and by not renewing existing licenses.

In 2013, the Indian Bureau of Mines (a government agency dealing with mining) was questioned in Parliament about whether they had conducted a study on the health effects in labourers engaged in asbestos mining. The agency had performed a science and technology project entitled “Study of Pollution Level in Asbestos Mines and Processing Plants in Rajasthan,” which recommended that restrictions on grant and renewal of mining leases and expansion of mining could be lifted subject to the implementation of safeguards on pollution levels in the work environment.^21^

The Government also commissioned the ‘National Study on Occupational Safety, Health and Working Environment in Asbestos-Cement Product Industries’,^24^ under the direction of the Directorate General Factory Advice Service and Labour Institutes, Ministry of Labour and Employment, Government of India, in 2019. The government reported in an answer in 2022, quoting this study, that 50 functional asbestos-cement industries were examined in the country. Of the 2,603 workers, 10 cases were found to be suspected cases of asbestos-related disorders. It further states that the concentration of airborne fibres in industries where inbuilt environmental control measures are in place and good practices followed, were found to be quite low compared to those units where such measures were unavailable. The study also found that the levels of asbestos fibres in 35 industries out of 50 were well within the permissible level of exposure, i.e., 0.1 fibre/cc. However, in 15 industries, the asbestos fibre concentration exceeded this, with values ranging from 0.185 to 0.400 fibre/cc. This study, which is the most recent, was presented in Parliament as a response to a question regarding whether any research had been carried out on the harmful effects of asbestos on human health.^3^ This study has some potential limitations, including the possibility that disease latency could be a factor, as the presence of disease may only be revealed decades after exposure. Furthermore, there appears to be no record of external peer review by an organisation outside the one conducting the study. Also, some experts have raised concerns about the safety of the permissible limit of 0.1 ­fibre/cc, as it has been documented elsewhere to be insufficiently protective and lacking necessary safeguards.

In this paper, we have provided an analysis of the government’s perspective regarding asbestos, its use and its impact on human health, as conveyed through its official statements in the Indian Parliament.^3^ To note, our paper only incorporates two external viewpoints that the Indian Government has not previously cited in its responses: the WHO’s assertion that all forms of asbestos, including chrysotile, have negative health consequences,^1^ and the position of a manufacturer of asbestos sheets who claimed that chrysotile is safe.^10^ The answers provided in Parliament demonstrate a remarkable consistency in the views of successive executive administrations regarding the dangers of asbestos (despite changes in government every 5 years). The overwhelming body of evidence (including data from the WHO and numerous other countries around the world) clearly documents the significant health hazards associated with asbestos, particularly in the context of cement product manufacturing. In light of our analysis, it may be concluded that India may be choosing to be an outlier when it comes to protecting workers in asbestos processing/product manufacturing plants, and heeding the impact on public health due to the continued use of asbestos. Instead, the government seems to defend the use of chrysotile. This has the potential to put the life of workers in the processing plants, and the people exposed indirectly to asbestos fibres, at risk. Given that the most prevalent use of asbestos in India is in the form of asbestos-cement sheets, which have a detrimental impact on the health of those who use them, it is crucial to prioritise demand-driven solutions to bring about meaningful change. This can be done by promoting alternative types of fibres and materials for roofing, as this will not only give the consumers an option, but also continue to provide a livelihood for the current asbestos sheet manufacturers and others who depend on its trade. Only demand-based pushes can impact the manufacture and import of asbestos fibres in India and across the world. Governments must build sustainable financial subsidy models and support alternative products for people using asbestos-cement sheets for housing. However, if this is not prioritised, then banning asbestos in all forms may be the only solution. Each asbestos-cement sheet that is produced and utilised poses a significant risk of disease throughout its entire life cycle, from mining and processing to installation, demolition and disposal. All those involved in these processes face a significant risk of exposure. Furthermore, individuals who occupy buildings constructed with asbestos-cement sheets are also vulnerable to inhaling hazardous asbestos fibres released into the air. Although industries can thrive through various means, human life can only be protected by avoiding dangerous materials such as asbestos.
